# Determination of Loss of Reinforcement Due to Corrosion through X-ray Computer Micro-Tomography

**DOI:** 10.3390/ma14040893

**Published:** 2021-02-13

**Authors:** Fernando França de Mendonça Filho, Oguzhan Copuroglu, Erik Schlangen, Branko Šavija

**Affiliations:** Materials & Environment, Faculty of Civil Engineering and Geosciences, Delft University of Technology, Stevinweg 1, 2628 CN Delft, The Netherlands; o.copuroglu@tudelft.nl (O.C.); Erik.Schlangen@tudelft.nl (E.S.); b.savija@tudelft.nl (B.Š.)

**Keywords:** X-ray tomography, reinforcement corrosion, non-destructive testing

## Abstract

X-ray computer scanning tomography (CT scan) is an increasingly more available technique, which has been applied to material sciences for years. Although most of its use is qualitative for gaining insights on material behavior, quantitative analysis for estimations of deterioration rates is possible. This paper describes an unbiased, straightforward method to determine the amount of reinforcement lost to corrosion through the use of X-ray tomography without the need to remove the concrete cover. Other methods of assessment such as gravimetric analysis, half-cell potential, resistivity of mortar cover, corrosion current, and scanning electron microscopy (SEM) are used in the same samples for comparison. While the electrical and electrochemical tests are valuable to describe the state of the samples, those demonstrated poor capacity of determining the stage of corrosion of the reinforcement in terms of amount of material lost. Electron microscopy could determine how much of the reinforcement corroded with high accuracy; however, these results are deficient in representativity, being based on a single plane of the steel. X-ray tomography, while suffering from sample size limitation, could provide quantitative information on the total volume of material lost for each sample with far higher accuracy than indirect techniques, which is significant for the forensic determination of remaining life service of structures.

## 1. Introduction

Corrosion of steel is a global problem with large financial implications. It is estimated that it represents 1% to 5% of a nation’s GDP, and in the United States alone its annual costs are estimated to be more than 250 billion dollars [1]. In order to reduce the costs, fundamental understanding of corrosion process accompanied by the development of mitigation and repair techniques is necessary. As concrete is the most widely used construction material, it is natural that considerable effort is put in improving corrosion resistance of its reinforcement [2], yet there is a number of uncertainties regarding the rate and reach of reactions standing in the way of accurate predictions.

One of the main challenges in studying the corrosion of reinforcement steel is that the process is directly affected by its environment. While it is possible to create simulated pore solutions to directly observe test samples [3], these neglect to take the influence of concretes microstructure into account. Further, tests performed in simulated pore solution might contain inaccurate estimations of pH and free alkalies [4] and often lead to non-representative results [5]

In order to study the reinforcement while still embedded in concrete, non-destructive testing (NDT) is often used. However, it usually provides qualitative assessments and lacks precision (e.g., half-cell potential and electrical resistivity) [6]. An alternative is to wait until corrosion starts and breaks the sample to analyze the damage on the surface. This introduces several problems as sample handling and conservation becomes an issue. Subsequently, sudden exposure to atmosphere can speed up reactions.

Recent advances in X-ray computer tomography contributed to the adoption of the technique in the field of cement and concrete research [7,8] and specifically, corrosion science [9,10,11,12,13,14,15]. The technique allows visualization of processes inside concrete without the need of fracturing the samples. In addition, image analysis grants three-dimensional quantitative information from the observations, in opposition to most techniques that require extrapolation from two-dimensional analysis.

While each of the aforementioned techniques has been used to study corrosion of steel embedded in concrete [16,17,18,19], the comparison of such techniques studying the same sample under the same conditions is fairly rare in literature. For this reason, this paper presents an assessment of the accuracy of micro-tomography against gravimetric and electrochemical analyses through comparison of plain and embedded steel bars in mortar exposed to saline solution until corrosion took place.

Plain bars were used for a direct measurement of loss of reinforcement due corrosion because of the lack of such validations in literature. This short experiment compares the sensitivity of the tomography with gravimetric analysis for small amounts of material loss. Next, mortar specimens exposed to chloride solution were monitored using electrical resistivity, half-cell potential, corrosion current, X-ray tomography, and scanning electron microscopy. The results were critically compared to evaluate usefulness in structure service life predictability.

## 2. Materials and Methods

### 2.1. Samples and Exposure

**Figure 1:**
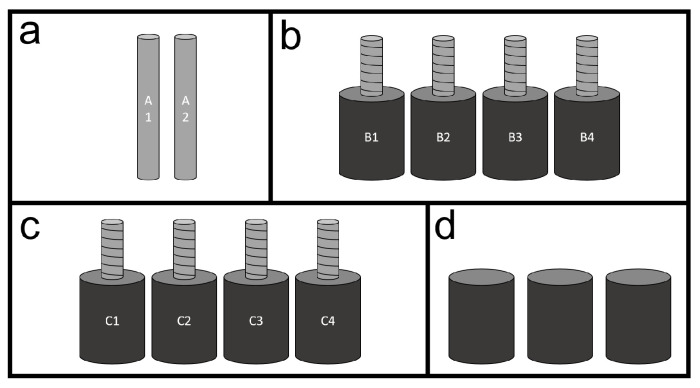
Sketch of different sample sets used for experiments: (**a**) Plain steel bars, (**b**) plain mortar cylinders, (**c**) mortar cylinders reinforced with ribbed steel bars, and (**d**) mortar cylinders reinforced with ribbed steel bars that were put in compression.

Four sample sets were used for the experiments, as sketched in Figure 1. The first set (assigned with the letter “a” in the figure) consists of plain steel bars and is described in the second paragraph of this section. The second and third sets (assigned with the letters “b” and “c” in the figure) consist of mortar samples reinforced with ribbed steel bars, these are described in the third paragraph of this section. The fourth set (assigned with the letter “d” in the figure) consists of plain mortar samples discussed in the fourth paragraph of this section.

For the gravimetric analysis, two 8 mm diameter and 50 mm height cylindrical steel bars were used. The bars (designated as A1 and A2) were exposed to a solution of 3.5 wt.% of sodium chloride for three months and their variation in mass was compared to the variation estimated by the micro-tomography. Prior to exposure, the bars were cleaned with concentrated hydrochloric acid for 3 min, then the mass was determined and subsequently a CT-scan was performed to register the initial state. During the exposure, the bars were kept in a laboratory environment with average temperature of 22 °C and 65% relative humidity. Afterwards, the bars went through mechanical cleaning, mass determination, and a second CT scan.

For the comparison with non-destructive tests, the second experimental set was composed by ribbed steel bars of 8 mm diameter and 70 mm height with 35 mm embedded in mortar. Ordinary Portland cement (CEM I 52.5 R) was used to cast the mortar cylinders using a water/cement ratio of 0.45. Standard sand (CEN-NORMSAND DIN EN 196-1) with a maximum aggregate size of 4 mm and tap water were used. Before casting, the steel bars were thoroughly cleaned both mechanically and with concentrated hydrochloric acid, so no rust was present. Each bar was fixed in the mold previous to casting. During casting, a small mechanical vibrator was coupled to the top of the bars to improve compaction of the mortar. The mortar was poured vertically, following the orientation of the steel. Eight reinforced cylinders of 24 mm diameter per 35 mm height were cast. Next, all samples were contained with plastic and kept in laboratory conditions for 24 h, followed by de-molding and storing in a 20 °C, >90% RH (fog) room for 28 days.

Three unreinforced mortar samples of the same mix were cast to determine the compressive strength of the mix. These three samples were tested at a fixed loading rate of 13.5 kN/s using a a servo-hydraulic mechanical press with maximum load capacity of 5000 kN and high stability from Matest.

From the reinforced mortar samples, four were studied without any loading (B1 to B4) and four were loaded in uniaxial compression up to 30% of the failure strength (C1 to C4). On one hand, it was chosen to load the samples so a state closer to practice could be studied. On the other hand, small samples were necessary to allow for good uniform sustained compression through the duration of this experiment. Thus, instead of large concrete samples with 16 mm rebars, the authors opted for mortar samples with 8 mm steel.

**Figure 2:**
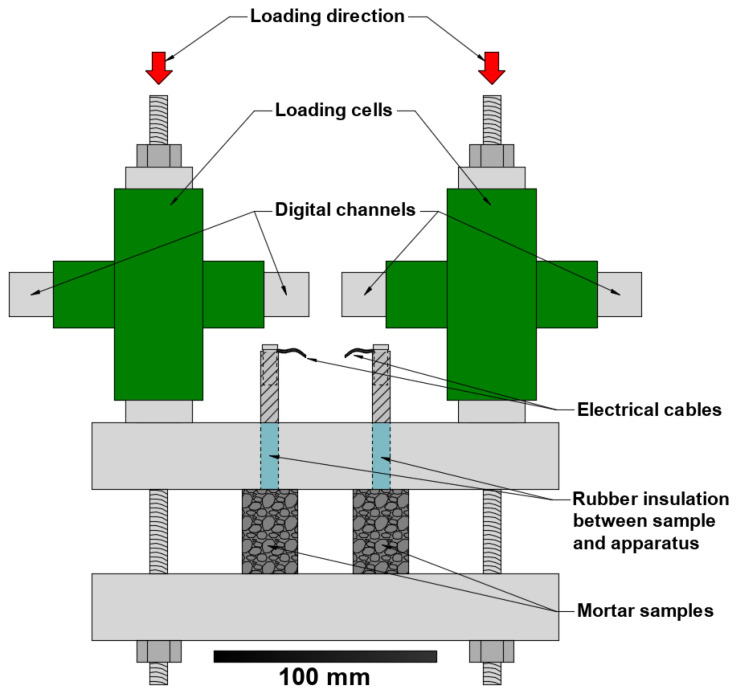
Experimental set-up for samples subjected to compressive loads.

The partial compression of samples C1 to C4 was achieved through the use of a couple of loading cells and a stainless steel set-up stiff enough to be considered rigid (Figure 2). All samples were then immersed in a 3.5 wt.% sodium chloride (NaCl) solution for 60 days in laboratory conditions (22 °C, 65% RH), and the level of the solution was slightly lower than the top of the mortar in order to avoid direct contact of salt with the reinforcement. During the period of exposure the solution was constantly renewed in order to keep NaCl concentration constant.

### 2.2. Electrical and Electrochemical Measurements

The half-cell potentials were measured according to the described procedure in ASTM C876 [20]. A standard calomel electrode (SCE) was used, and hourly registrations were performed using one digital channel per sample. The convention adopted is the one suggested in ASTM C876 (summarized in Table 1).

Measurements of electrical resistivity were taken weekly through a standard multimeter using a titanium mesh as cathode in contact with each sample and a sponge around the system to secure moisture of chloride solution. With the steel bars acting as anode, the equipment measured the electrical resistance generated by the concrete cover.

The corrosion current was indirectly determined by means of linear polarization resistance using a multicorr device connected the steel–titanium system with the SCE embedded in the solution. The measurements were also done weekly. A detailed description of the procedure can be found elsewhere [21].

### 2.3. X-ray Micro-Tomography

All micro-tomography analyses were performed using a Phoenix Nanotom X-ray CT scanner. The machine consists of a transmission type X-ray tube, a sample stage, and a 3072 × 2400 flat panel detector. The transmission target uses a tungsten filament and possesses a maximum accelerating voltage of 180 kV. The acquisitions were performed at 120 kV, with a step size of 0.25°, an exposure of 500 ms, and averaging of two images. A geometrical magnification of 3.333 was used resulting in a spatial resolution of 15 μm. Reconstruction was done through datos veloCt, the software provided by the equipment manufacturer.

**Figure 3:**
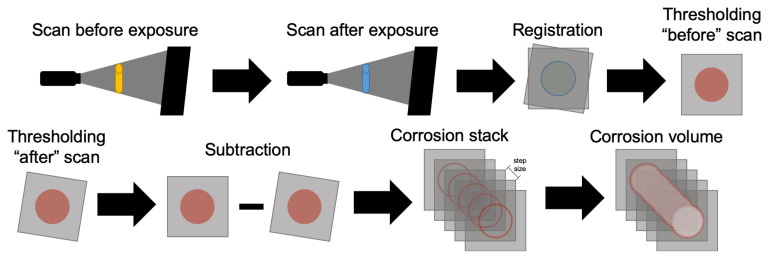
Schematic representation of the method used for determination of material loss through CT scans.

For samples A1 and A2, the postprocessing of the images used the freeware Fiji [22]. To account for slight misalignment between the scans performed before and after exposure, rigid registration was carried out. Max entropy threshold [23] was used for segmentation, followed by the subtraction of the second scan out of the first (scheme in Figure 3). The result was a binary stack containing only lost material. The interest area of each image was computed and multiplied by the step size to give the results in volume.

For samples B1 to B4 and C1 to C4, the same equipment and procedure were used, but a higher geometrical magnification allowed for the slightly improved pixel size of 12 μm.

### 2.4. Scanning Electron Microscopy

The samples for corrosion in mortar were later cut in order to be analyzed in an electron microscope. Samples B2 to B4 and C2 to C4 were selected to carry this analysis. In order to avoid the washing out of chlorides by water, the samples were dried in an oven at 40 °C and cut using ethanol as cooling agent. Next, impregnation of the surface of the samples with a low-viscosity epoxy resin was carried under vacuum. Each sample was then ground and polished until the surface achieved satisfactory flatness. The polished surfaces were covered with a 10 nm layer of conducting carbon coating using a Leica EM CEDO030 carbon evaporator.

Following, each of these samples was studied with BSE (backscattered electron) imaging and energy dispersive X-ray spectroscopy (EDS). The microscope used was a QUANTA^TM^ FEG 650 from Thermo Scientific^TM^ equipped with a field emission gun and circular backscatter detector. A Philips XL30 was used for the EDS analyses with a take-off angle of 35.3° and sample to detector distance was 10 mm. The samples were analyzed under high vacuum. The detector was a SUTW (sapphire), the accelerating voltage was set at 15 kV, the calibrated resolution was 132 eV, and the deadtime was kept at approximately 10%. Additional details regarding the spectra correction for elemental quantification can be found elsewhere [24].

## 3. Results

### 3.1. Corrosion in Solution

**Figure 4:**
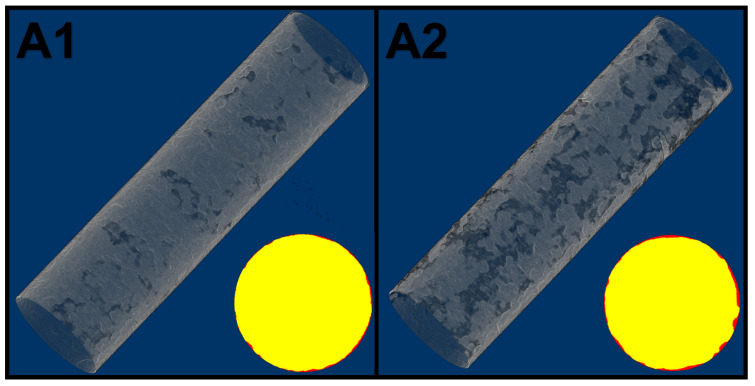
Volume of steel lost to corrosion (in gray) from samples (**A1**) and (**A2**), in the bottom there are cross sections with steel in yellow and corrosion products in red.

The results of the registration process on the tomography of the plain bars can be seen in Figure 4. The loss of volume of the samples is presented in Table 2. The mass values of the gravimetric analysis were converted to volume in function of the measured density of each samples. The agreement between techniques was very good, especially considering the small scale of the study. Both samples went through general corrosion, the view in Figure 4 might be misleading as A1 seems to have more distributed loss of material, however, upon cross section analysis it is noticeable that A2, although more spaced, has deeper patches of damage. The slight misfit between the techniques is attributed to the necessity of averaging the space between slices of the tomography stacks.

### 3.2. Corrosion in Mortar

#### 3.2.1. Electrical Testing

**Figure 5:**
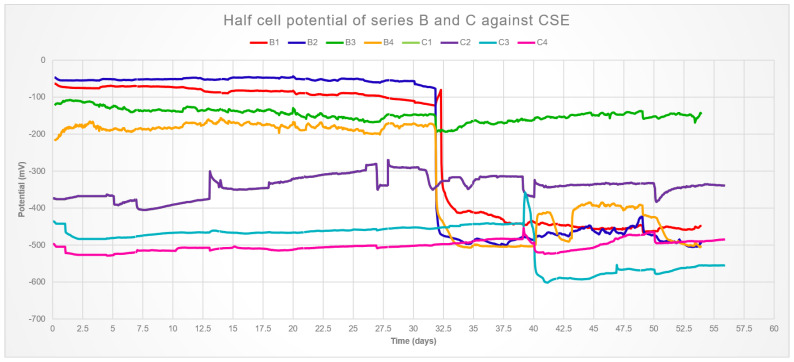
Electrical potential of samples B1 to B4.

The results for the electrical potential over time are show in Figure 5. Although a calomel electrode was used, the results were converted to their copper–copper sulfate respective so Table 1 could be used. An evident distinction is observed between series B and C, the unloaded samples started with values above the threshold and corrosion seems to take place after 32 days of immersion, with the exception of sample B3 that has not entered the corrosion zone.

The average result for compressive strength at 28 days of the mix used was 32.26 MPa, and therefore the samples were loaded accordingly. These samples were in the corrosion zone from the beginning. This is attributed to crack development during loading, which was visible only in samples C1 and C2. However, it was found that cracks as small as 80 μm are enough to allow for rapid diffusion and corrosion initiation [25], thus it is reasonable to assume micro-cracking in samples C3 and C4 as well.

Samples C1 and C2 present nearly the same signals, the difference in the raw data is an order of magnitude smaller than what is visible in the graph, thus it was assumed that the bars were electrically connected. Most likely, the bar positioning with respect to the bottom of the mortar was too close to the mold during casting, which could favor cracking at the bottom of the samples. This would allow a connection through the loading set-up. Because the device used for loading is made of stainless steel, therefore the whole system would generate a single electrical output.

Resistivity and corrosion rate are presented in Table 3 and Table 4. The resistivity of samples B1 and B2 matches well the potential results, the values presented the same drop in the fifth week. Values for all other samples remained constant, which is in agreement with potential results with the notable exception of B4, that has a variation too great to allow for a trend. Micro-cracking would also explain the two orders of magnitude difference in the resistivity of the loaded samples, values so low indicate that resistivity is not a proper parameter to evaluate those samples [26]. Nevertheless, C1 is clearly more noble with respect to C2, therefore probably the cathode in the electrical pair hypothesized. This means that C2 is most likely acting as a sacrificial anode for the other steel bar.

The corrosion currents varied from negligible to moderate for samples B1, B2, and B4, even after the forth week. Conversely, sample B3 had a sharp increase in current in the fifth week. C1 and C2 had a much higher current, characteristic of an electrical bridge between them that kept stable during the full period of the experiment. Likewise, the values for samples C3 and C4 did not change. It is not inconsistent for bars B1, B2, and B4 to present such values, as the potential and resistivity are merely indicatives of the environment surrounding the steel and do not give information on the actual corrosion kinetics [27]. This is also exemplified by the loaded samples, which presented similar values of resistivity and half-cell potential, but very different corrosion rates. The behavior of sample B3, on the other hand, was unexpected. The presence of high corrosion currents during relatively high potentials and resistivity is rare.

#### 3.2.2. Tomographic Analysis

A reference scan of each sample was performed before Cl${}^{-}$ exposure. After eight weeks of exposure, the compression set-up was disassembled and a second tomography was performed on the samples. Corrosion micro-pits usually form with a depth and diameter between 100 μm and 150 μm, respectively. Outliers can start as small as 20 μm [28], which justifies the chosen resolution for this experiment.

**Figure 6:**
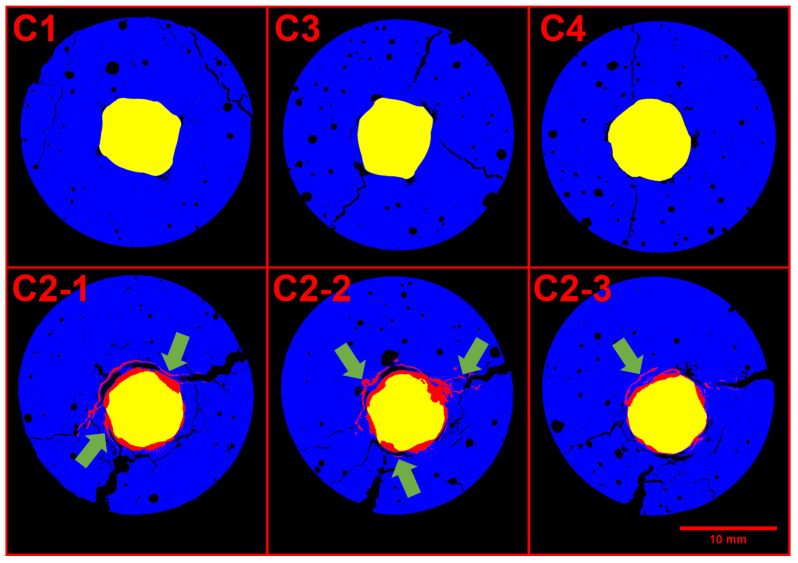
Cross section of loaded samples, the mortar phase is displayed in blue, pores and cracks in black, the steel is in yellow, and the corrosion products are shown in red. The green arrows show cracked zones filled with corrosion products.

The mortar portion of the samples was undamaged in all unloaded samples, but presented cracking in the loaded ones, Figure 6 shows the cracking on each sample. Three slices at increasing depth are shown of C2 given its higher variability depending o height.

The reference scans did not contain significant cracks; therefore, such cracks happened during the exposure period. Sample C1 had minor cracking on the surface, but nothing close to reinforcement. Samples C3 and C4 had cracks going from the surface until reinforcement with an average size of 0.2 mm and 0.1 mm, respectively. These observations agree with the previous assumptions that C1 was acting as cathode and C2 as anode, given the obvious ease of chloride access to C2. Further, the confirmation of microcracking in C3 and C4 explains the electrical results. Still, C3 and C4 did not display considerable rust formation, thus, the cracking is most likely caused by the mechanical loading.

Specimen C2, on the other hand, might have a combination of the loading plus the stress from rust formation as responsible for the cracks. There were two main fractures in the sample with an average opening larger than 0.8 mm that correlate well with the corrosion products (as pointed in Figure 6).

**Figure 7:**
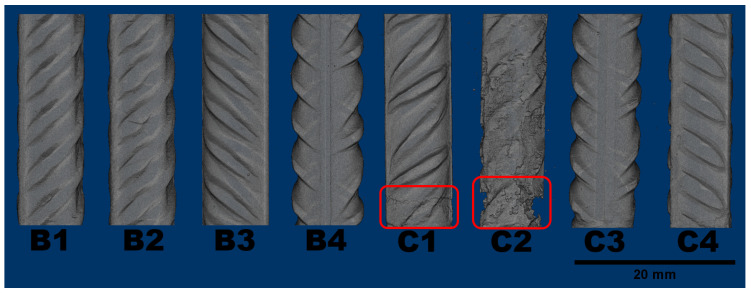
Reconstruction of the remaining sound steel in each sample after 60 days of exposure.

Regarding the steel phase, detailed volumetric reconstructions are displayed in Figure 7. Uniform corrosion is often hard to notice in the initial state because of the small change in cross section. The notable exceptions are samples C1 and C2 that rather suffered localized corrosion at the bottom (marked in red on Figure 7). It should be noted that the deterioration of both samples is present at the bottom, where the connection was presumed.

Localized corrosion is particularly dangerous because of the speed in which diminishes the load capacity. Sample C2 poses as a prime example, after only eight weeks, it has little more than half its original cross section at critical height. Even if another sample had lost the same amount of reinforcement through uniform corrosion, the loss in cross section would be much smaller, which highlights the importance of knowing the corrosion location. Table 5 shows the changes in volume for each steel bar computed through tomography and the results of corrosion current.

#### 3.2.3. Electron Microscopy

**Figure 8:**
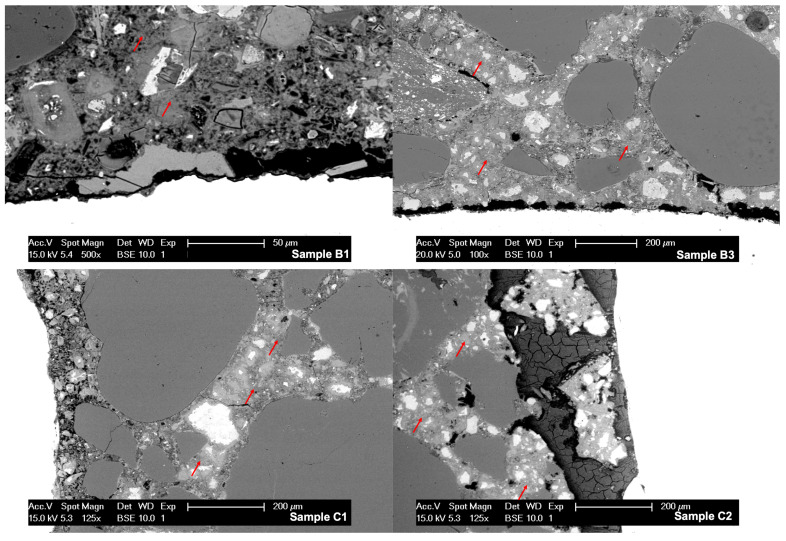
Micrographs of samples B1, B3, C1, and C2. Red arrows indicate typical spots where EDS analysis was carried. The porous zone between the mortar and the steel in samples B1 and C1 can be seen, the morphology of corrosion products in sample C2 can be appreciated in dark gray.

An average of 50 EDS spectra were collected per sample. These were pointed at C-S-H preferentially, so the chloride content could be quantified and atomic ratios were estimated. Typical locations for the analysis can be seen in Figure 8 marked by red arrows. In the same image it is also possible to observe high porosity zones in samples B1, B3, and C1, usually between 50 μm and 200 μm from the steel. Sample C2 presented corrosion products between mortar and the reinforcement.

The quantification performed by EDS analysis showed consistent concentration of chlorides in all studied samples. The amount per sample (averaged over 8 mm) is shown in Table 6. Concentrations were very high, which is to be expected for two reasons: the studied was carried past the point at which most samples started corrosion according to the electrical potential results, and this technique reports total chloride content rather than only free chlorides. The choice of working with total chloride content was due to remaining controversy in literature on the harmlessness of bound chlorides [29,30].

**Figure 9:**
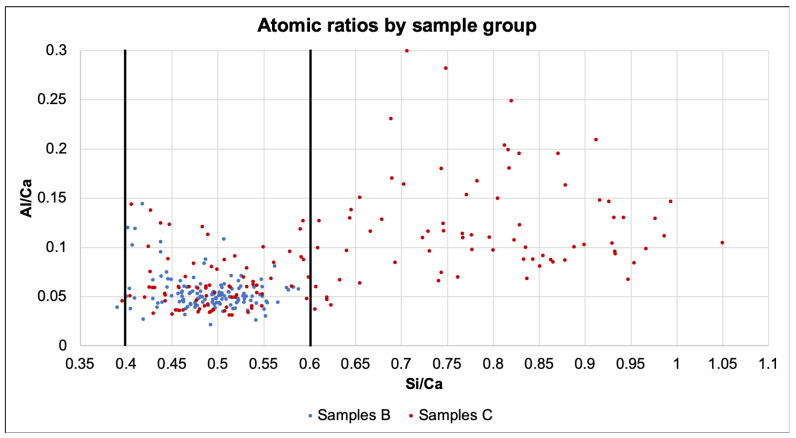
Atomic ratios of samples separated by series.

The atomic ratios are presented in Figure 9. While all samples of series B present a Si/Ca ratio between 0.4 and 0.6, more than half of the other samples display a much higher ratio, close to 1.0. Because the chloride solution was changed weekly, the chemical shock might have caused leaching on the samples. As specimens C1 to C4 had microcracking from the loading, the solution could arrive farther in less time, facilitating the leaching. Additionally, Rougelot et al. [31] found that leaching can further cause micro-cracking, starting a positive feedback loop. Whether the same phenomena can be expected in full scale structures or has been emphasized due the small size of the samples is not clear and requires future investigations.

**Figure 10:**
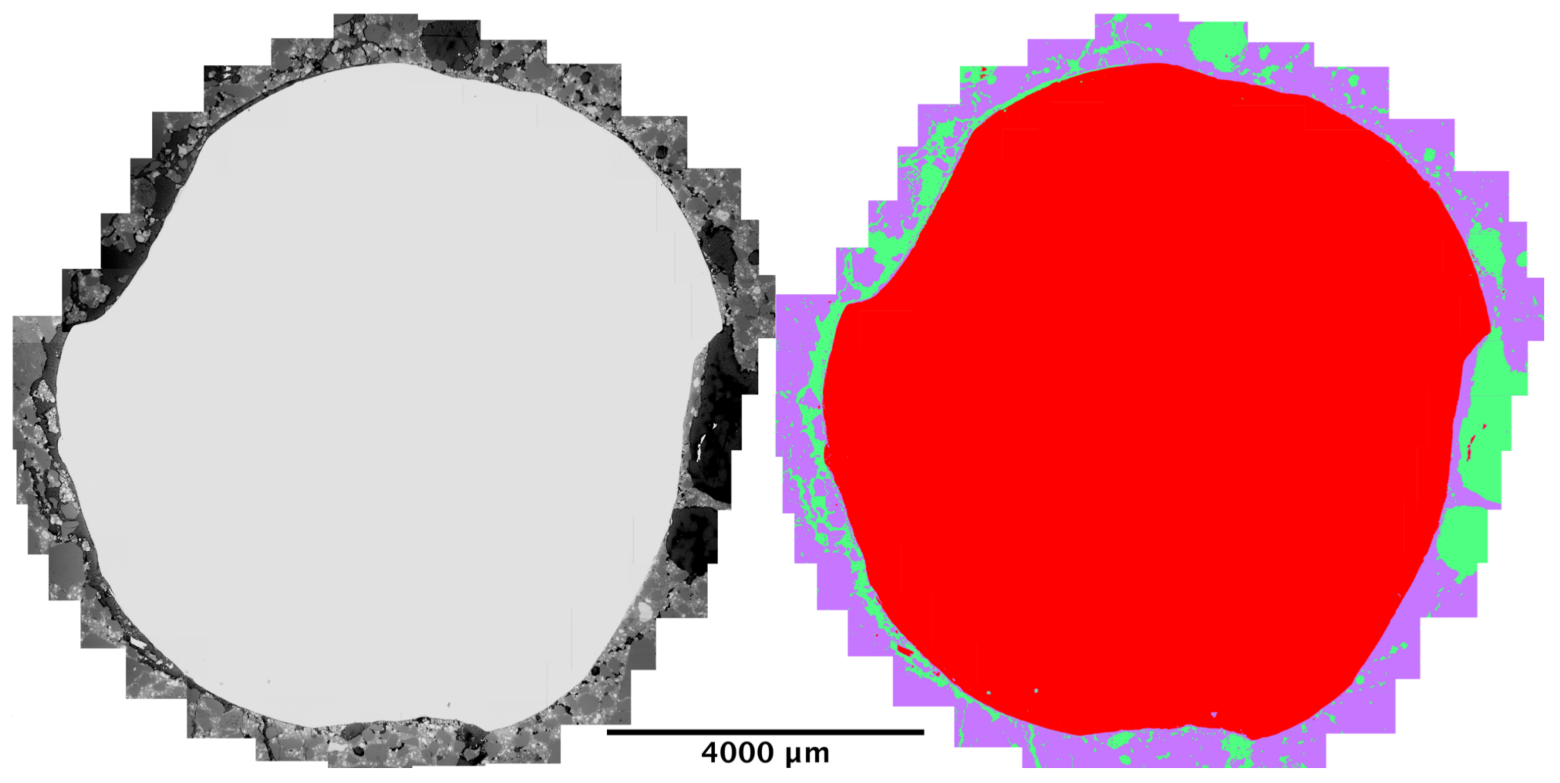
(**Left**) Stitching of 34 electron micrographs to obtain the region around the steel bar on sample C2. (**Right**) Segmented version of picture on the left, steel is in red, mortar in purple, and rust in green.

Because sample C2 presented the highest amount of corrosion, it was decided to carry supplementary analysis on it. The region around the reinforcement (between 500 μm to 1 mm from the steel) was thoroughly scanned and a mosaic of 34 micrographs was assembled, this is justified by the importance and variability of scales of effect on the steel–concrete interface [32]. Afterwards, segmentation was performed to classify the components into steel, mortar or corrosion products (shown in Figure 10).

## 4. Discussion

### 4.1. Estimation of Damage Initiation and Amount

**Figure 11:**
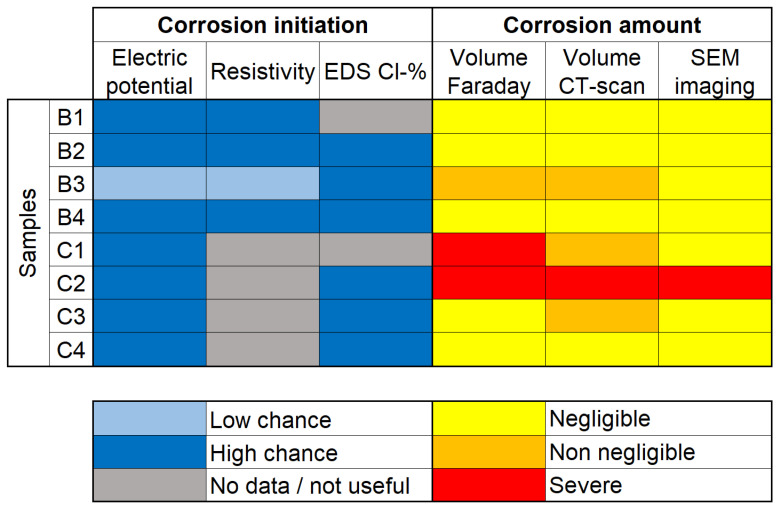
Comparison of different techniques used to determine corrosion initiation and amount.

Structures are tested in order to aid the estimation of their integrity. In the context of corrosion by exposure of the reinforcement to an environment with high amounts of chloride, the main information desired is whether corrosion has started and, if so, how much has taken place. Figure 11 displays a simplified summary of the conclusions derived from individual tests performed in this study.

Both resistivity and EDS analysis of Cl${}^{-}$ content are indirect techniques that give information on the environment surrounding the reinforcement, rather than the state of the reinforcement itself. Electrical potential is a somewhat more direct method, as the electrodes are directly connected to the reinforcement. Nevertheless, these can only tell if the conditions for corrosion are present, not if corrosion has in fact initiated. This can be seen in Figure 11 by noting that although samples B2 and B4 had a “high chance” of corrosion found by all three methods, these samples have not presented noticeable amounts of corrosion. Still, it is possible that, had the experiment continued for longer, the samples could start corroding in a higher rate.

On the other hand, sample B3 presented “low chance” of corrosion with both electrical potential and resistivity, only the EDS analysis pointed to high risk scenario. This is remarkable because sample B3 was the only non-loaded sample which corroded considerably. A possible reason for that would lie in the fact that EDS analysis is actually a semi-destructive technique, which requires the extraction of small amounts of material for analysis, thus having more direct measurements.

In the case of tests pointing to corrosion initiation, it is often advisable to perform further experiments in order to determine how much has been lost to corrosion. The detection of corrosion products has a high precision with SEM imaging; however, the estimation of corrosion amount is poor for two main reasons: the limitation of analysis of a single “slice” per sample and the high magnification, which makes analysis of big areas restrictively time demanding. Because of this, SEM imaging is often use as a support tool, rather than an exploratory method for corrosion amount. It is also possible to notice this by looking at the right side of Figure 11, in which the selected slices only showed corrosion products in sample C2.

By contrast, both CT scanning and Faraday law estimations based on corrosion current can evaluate large areas of samples at once with enough sensitivity to detect corrosion. Both techniques show good agreement (Table 5), although some caveats appear for the estimations with Faraday law. The corrosion volume in five out of eight samples was underestimated using the Faraday law and the value for sample C1 was significantly overestimated because of the electrical connection on the C1–C2 pair. As the tomography looks at the full volume of the sample, detection of corrosion is only limited by its resolution.

### 4.2. Structural Integrity Assessment

Inspections are often limited in time and amount of samples, which requires engineers to find ways of making informed decisions with little information. Thus, choosing the optimal methods are key for structural integrity assessment.

Corrosion currents are not constantly monitored, usually punctual measurements are performed sporadically. As this information is used to define a rate of corrosion, the averaging of the value over long periods of time often leads to too large over- or underestimations. Additionally, if a bar is completely corroded, the current will be very low, also leading to erroneous conclusions.

In the case of microscopy, the type of analysis is valuable for additional information on both, thermodynamics and kinetics of corrosion, as it can provide the distribution and composition of corrosion products (see Figure 10). However, because this technique still requires many hours of specialized labor, it is often performed for a single cross section, which is hardly representative. In the case of the present study, it was later determined that for an accurate distribution of the corrosion products, a bigger area of the sample would be necessary, requiring many hundreds of micrographs. In addition, the stitching of big amounts of high quality pictures generates image files of several tens of gigabytes, which can demand considerable computation power during image analysis.

In this context, CT scan has showed fast and reliable detection power, which can aid in the assessment of structures without requiring many resources. Notably, the technique is limited by the amount of cores possible to extract from a building and the resolution achieved in function of the sample size. Yet, it seems to have a considerably higher accuracy than electrochemical methods and higher representativity than 2-D methods.

## 5. Conclusions

A number of tests were used to detect and quantify corrosion in comparison to tomography. Gravimetric analysis was used to secure accuracy of the technique before analysis against indirect methods and presented strong agreement. The results of half-cell potential and resistivity measurements can only give information regarding the likeability of corrosion presence, not the rate at which is happening. Thus, those should be used as guides to which areas deserve further studies. In this context, the tests failed to warn about the slow corrosion in sample B3 and gave somewhat misleading results for samples B1, B2, and B4.

The microscopic analysis provided valuable complementary information, but its destructive nature and very small area of analysis are appreciable set backs to the routine use of it. This is a technique better reserved for anomalous samples deemed to require further investigation.

The tomography scans showed the actual damage present in the samples, which agreed well with estimations based on corrosion currents with exception of the bars electrically linked. As in practice most bars are connected to others through shear reinforcement, this can lead to troublesome assumptions.

From the scientific point of view, tomographic analysis can produce an abundance of information that simply cannot be matched by other non-destructive techniques. From the practical standpoint, the visualization granted by this technique allows to identify types of corrosion in place and fit it to standard models with μm precision. Such fitting permits to estimate when corrosion first took place, the state of the structure and time in which further action is necessary.

Additionally, an appropriate setup could provide validation to models trying to simulate steel corrosion in cracked concrete for both, the mechanical and transport functions. The authors judge the potential of coupling experimental data provided by this technique with numerical models a big advantage.

Yet, computer tomography can only be used as a non-destructive technique for small movable samples at present. Actual field samples can only be analyzed through coring. Therefore, the strength of the technique is in the combination with other electrical and electrochemical methods mentioned herein. Faster and simpler techniques, like resistivity mapping, could point to key points to be studied in a full structure. Then, a much smaller volume would be analyzed through micro-tomography, generating very detailed information.

## Figures and Tables

**Table 1:** Guideline to corrosion detection according to ASTM C876 using a copper–copper sulfate electrode (CSE).

Sample Condition	Potential (mV)
Passive (>95% certainty)	0 to −200
Intermediate range	−200 to −350
Corroding (>95% certainty)	−350 to −600

**Table 2:** Volume loss to corrosion (mm${}^{3}$) by gravimetric and tomographic analysis.

Sample	Gravimetric Analysis	Tomographic Analysis
A1	59.74 ± 0.01	55.06 ± 0.05
A2	62.46 ± 0.12	62.75 ± 0.06

**Table 3:** Resistivity of samples (kΩcm).

Week	B1	B2	B3	B4	C1	C2	C3	C4
1	146	151	152	1	1.5	0.4	0.3	0.5
2	115	170	105	66	1.1	0.7	0.1	0.6
3	156	161	120	33	1.1	0.3	0.1	0.1
4	151	153	105	49	1.4	0.4	0.4	0.8
5	41	44	115	43	1.5	0.7	0.1	0.2
6	50	25	165	23	1.3	0.5	0.8	0.6
7	51	50	122	56	1.0	0.2	0.8	0.1
8	46	30	108	32	0.9	0.2	0.4	0.1

**Table 4:** Corrosion rate of samples (μm/year).

Week	B1	B2	B3	B4	C1	C2	C3	C4
1	0.2	0.0	0.0	0.1	1109	1180	51	47
2	9.9	24.3	0.2	0.0	847	915	52	46
3	1.8	9.9	0.1	3.4	578	764	46	42
4	2.1	4.7	0.3	0.3	1198	1190	43	38
5	4.0	8.4	189	0.0	1019	1049	41	32
6	3.3	8.2	204	0.0	1163	1195	48	37
7	5.9	5.9	201	3.9	1292	1148	96	45
8	9.3	6.1	163	0.0	1103	1080	78	30

**Table 5:** Material loss by each technique (in mm${}^{3}$).

Sample	By Tomography	By Faraday Law
B1	5.3	0.6
B2	0.6	1.2
B3	29.9	12.8
B4	5.5	0.1
C1	24.3	140.2
C2	165.5	143.7
C3	13.1	7.7
C4	0.5	5.3

**Table 6:** Amount of chloride quantified through energy dispersive X-ray spectroscopy (EDS) analysis.

Sample	Cl${}^{-}$ (%)
B2	5.27 ± 0.19
B3	4.69 ± 0.17
B4	3.66 ± 0.19
C2	4.66 ± 0.29
C3	4.78 ± 0.21
C4	5.02 ± 0.25

## Data Availability

Data sharing is not applicable to this article.

## Abbreviations

The following abbreviations are used in this manuscript:

EDS	Energy dispersive X-ray spectroscopy
CT scan	Computer scanning tomography
SEM	Scanning Electron Microscopy
SCE	Standard calomel electrode
CSE	Copper–copper sulfate electrode
